# New Supercapacitors Based on the Synergetic Redox Effect between Electrode and Electrolyte

**DOI:** 10.3390/ma9090734

**Published:** 2016-08-29

**Authors:** You Zhang, Xiuguo Cui, Lei Zu, Xiaomin Cai, Yang Liu, Xiaodong Wang, Huiqin Lian

**Affiliations:** 1 School of Material Science and Engineering, Beijing Institute of Petrochemical Technology, Beijing 102617, China; 2014210480@buct.edu.cn (Y.Z.); zulei@bipt.edu.cn (L.Z.); 13522433853@163.com (X.C.); 2 State Key Laboratory of Organic-Inorganic Composites, Beijing University of Chemical Technology, Beijing 100029, China; wangxd@mail.buct.edu.cn; 3 Beijing Key Laboratory of Specialty Elastomer Composite Materials, Beijing Institute of Petrochemical Technology, Beijing 102617, China; yang.liu@bipt.edu.cn

**Keywords:** supercapacitor, redox electrolyte, polyanilline, multi-wall carbon nanotube, synergistic effect

## Abstract

Redox electrolytes can provide significant enhancement of capacitance for supercapacitors. However, more important promotion comes from the synergetic effect and matching between the electrode and electrolyte. Herein, we report a novel electrochemical system consisted of a polyanilline/carbon nanotube composite redox electrode and a hydroquinone (HQ) redox electrolyte, which exhibits a specific capacitance of 7926 F/g in a three-electrode system when the concentration of HQ in H_2_SO_4_ aqueous electrolyte is 2 mol/L, and the maximum energy density of 114 Wh/kg in two-electrode symmetric configuration. Moreover, the specific capacitance retention of 96% after 1000 galvanostatic charge/discharge cycles proves an excellent cyclic stability. These ultrahigh performances of the supercapacitor are attributed to the synergistic effect both in redox polyanilline-based electrolyte and the redox hydroquinone electrode.

## 1. Introduction

The growth in hybrid electric vehicles, uninterruptible power, and mobile electronic devices markets promotes development of electrochemical energy storage systems. Supercapacitors (SC) can provide higher power density and have a longer cyclic life than ion batteries, although they suffer from relatively low energy density [1,2,3]. In order to reduce the gap of the energy density between SC and ion batteries, significant research on SC has been focused on the enhancement of specific capacitance and working voltage, since these properties have positive correlation with the energy density [4,5]. The preparation of novel electrode materials with new structures and components is an effective way to archive higher capacitance [1,6], and utilizing organic or ionic liquid electrolytes and asymmetric electrode configurations or hybrid integration can extend the working voltage window of the SC [7,8,9,10].

Recently, some special electrolytes containing redox compound have been employed as active electrolytes, and combined with carbonaceous electrodes for improving the performance of SC [11,12,13,14]. These redox compounds not only are directly involved in the electron transfer redox reaction on the electrode-electrolyte interface, but can also synchronously improve the ionic conductivity of the electrolyte. Li et al. have used FeSO_4_ and CuSO_4_ as redox additives to ameliorate performance of SC, and a higher specific capacity of 223 mAh/g has been obtained [15]. Frackowiak’s group has reported that SC based on an electrochemical combination of a carbon electrode and a KI-H_2_SO_4_ electrolyte provided 1840 F/g of maximum capacitance [14]. Ji et al. have prepared a SC with a cell voltage window of 1.6 V and energy density of 7.1 Wh/kg by uniting KI-KOH redox electrolyte and a carbon-based electrode [16]. Mai et al. have obtained 4700 F/g of capacitance from the cyclic voltammogram measurement in a three-electrode configuration consisting of CuCl_2_-HNO_3_ redox electrolyte and a porous carbon microsphere electrode [17]. Additionally, the redox reaction between hydroquinone (HQ) and quinine (Q) can yield a pseudocapacitive effect, thus the addition of HQ to an H_2_SO_4_ electrolyte caused a significant increase in the capacitance values for all of the carbon materials tested, and the maximum specific capacitance in a three-electrode system reached 5017 F/g [18,19]. In the mentioned reports, most of the electrochemical systems consist of carbon-based electrodes and redox electrolyte, whose capacitance is benefits from the low electric double-layer capacitance and high pseudocapacitance redox electrolyte. Theoretically, an optimal electrochemical system should be a combination of a redox material electrode and a redox electrolyte, which should be able to provide a double synergistic effect for SC performance than the reported combination of carbonaceous electrode–redox electrolyte. Unfortunately, until now, there are few reports about this kind of electrochemical combination.

Polyaniline generally possesses an excellent pseudocapacitance property as the electrode of supercapacitors. However, due to the single pseudocapacitance effect, the supercapacitors could not obtain a sufficient specific capacitance and energy density for some special applications. In our previous studies, we have firstly reported a significant interaction that existed among polyaniline and iodide ions in various electrochemical systems [20,21,22], including mesoporous manganese oxide/polyaniline-KI and mesoporous silica/polyaniline electrode-KI. However, because of insufficiency in electric conductivity and matching between the electrode and electrolyte, a synergetic redox effect was not fully developed, and the maximum specific capacity was limited to under 1800 F/g.

Inspired by these works, in this paper, we prepared a redox composite electrode of polyaniline and carbon nanotubes by in situ polymerization of aniline on the surface of the carbon nanotube, and then we deeply investigated the synergetic redox interaction between polyaniline-carbon nanotube and HQ in acidic aqueous electrolyte. By virtue of this extraordinary interaction, the electrochemical properties of the supercapacitor were greatly improved. Notably, the specific capacitance is 1.58 times of the highest value of redox electrolyte systems in past reports.

## 2. Experimental Section

### 2.1. Materials

Multi-walled carbon nanotubes (MWCNTs) were obtained from CNano Technology Co., Ltd., Beijing, China. Aniline (AN), ammoniumpersulfate (APS), and hydroquinone (HQ) were purchased from Aladdin Co., Ltd., Shanghai, China.

### 2.2. Modification of MWCNTs

MWCNTs were treated by a H_2_SO_4_/HNO_3_ mixture as reported in [23]. In a typical procedure, 0.2 g MWCNTs were added into a mixture of 75% sulfuric acid (3 mol/L) and 25% nitric acid (3 mol/L), and then sonicated for 30 min, followed by refluxing at 120 °C for 12 h. The resulting sample was filtrated and diluted by de-ionized water until neutral. Then, the sample was dried at 60 °C under vacuum for 24 h. The functional modified MWCNT was abbreviated to F-MWCNT.

### 2.3. Preparation of PANI/F-MWCNTs

The PANI/F-MWCNTs was prepared as the following method [23]: a mixture consisted of 0.25 g AN, 0.03 g F-MWCNTs, and 80 mL HCl aqueous solution (1.5 mol/L) was sonicated for 30 min, and then was stirred at 4 °C for another 30 min. After that, 20 mL APS aqueous solution (0.12 mol/L) was put into the fore-mentioned mixture and stirred at 4 °C for another 12 h. The resulting sample was filtered and washed with de-ionized water for several times until neutral. Lastly the sample was dried at 60 °C under vacuum for 24 h.

## 3. Characterization

### 3.1. Structure and Composition Characterization

Scanning electron microscopy (SEM) measurement was carried out in a COXEM-20 microscope (COXEM, Daejeon, Korea) at 20 kV. X-ray diffraction (XRD) patterns were obtained with a Bruker D8 diffractometer (BRUKER AXS, Berlin, Germany) in reflection mode using Cu Kα = 0.154 nm with a voltage of 40 kV. Infrared spectroscopy (IR) analyses were accomplished on a Thermal Nicolet infrared spectrometer (Thermo Fisher Scientific Inc., Waltham, MA, USA).

### 3.2. Electrochemical Characterization

The electrochemical measurements were tested mainly by a standard three-electrode cell; synchronously, the two-electrode symmetric electrochemical system was tested for a deeper exploration of the PANI/F-WMCNTs and HQ system. In details, the working electrode was composed of PANI/F-WMCNTs (80 wt. %), polyvinylidenefluoride PVDF (10 wt. %) and acetylene black (10 wt. %). The electrolytes were composed of 1 mol/L H_2_SO_4_ with different concentrations of HQ aqueous solution (1, 2, 3, and 4 mol/L). A saturated calomel electrode (SCE) and a Pt foil electrode were used as the reference electrode and counter electrode, respectively. The cyclic voltammetry (CV) tests were performed from −0.6 V to 0.8 V (vs. SCE) with different scan rates (1, 2, 5, and 10 mV/s). The galvanostatic charge/discharge (GCD) analyses were performed in a potential range of −0.4 to 0.7 V in the standard three-electrode cell and it is set to −0.8 to 1.2 V for the two-electrode symmetric electrochemical system (vs. SCE) at different current density (0.2, 0.5, 1, 2, and 5 A/g). Electrochemical impedance spectroscopy (EIS) measurements were carried out under open circuit conditions over a frequency region from 0.01 Hz–100 kHz by applying an AC signal of 5 mV in amplitude throughout the test. The CV, GCD, and EIS tests were all tested on a CHI660D electrochemical workstation (Chenhua Co., Shanghai, China).

The specific capacitance (*C*, F/g), equivalent series resistance (ESR), energy density (*E*, Wh/kg) and power density (*P*, W/kg), were calculated according to the following equations [17]:

(1) $$Cs=\frac{2i_{m}\int \Delta Vdt}{\Delta V^{2}}$$

(2) $$ESR=\frac{iR_{drop}}{2I}$$

(3) $$E=\frac{1000\cdot Cs\cdot \Delta V^{2}}{3600\times 2}$$

(4) $$P=\frac{3600E}{t}$$

where *Cs* is the specific capacitance (F/g), Δ*t* is the discharge time (s), *I* is the current loaded (A), Δ*V* is the potential window during the discharge process, *m* is the mass of electrochemical active material in electrode (g), and iRdrop is the potential drop.

## 4. Results and Discussion

**Figure 1:**
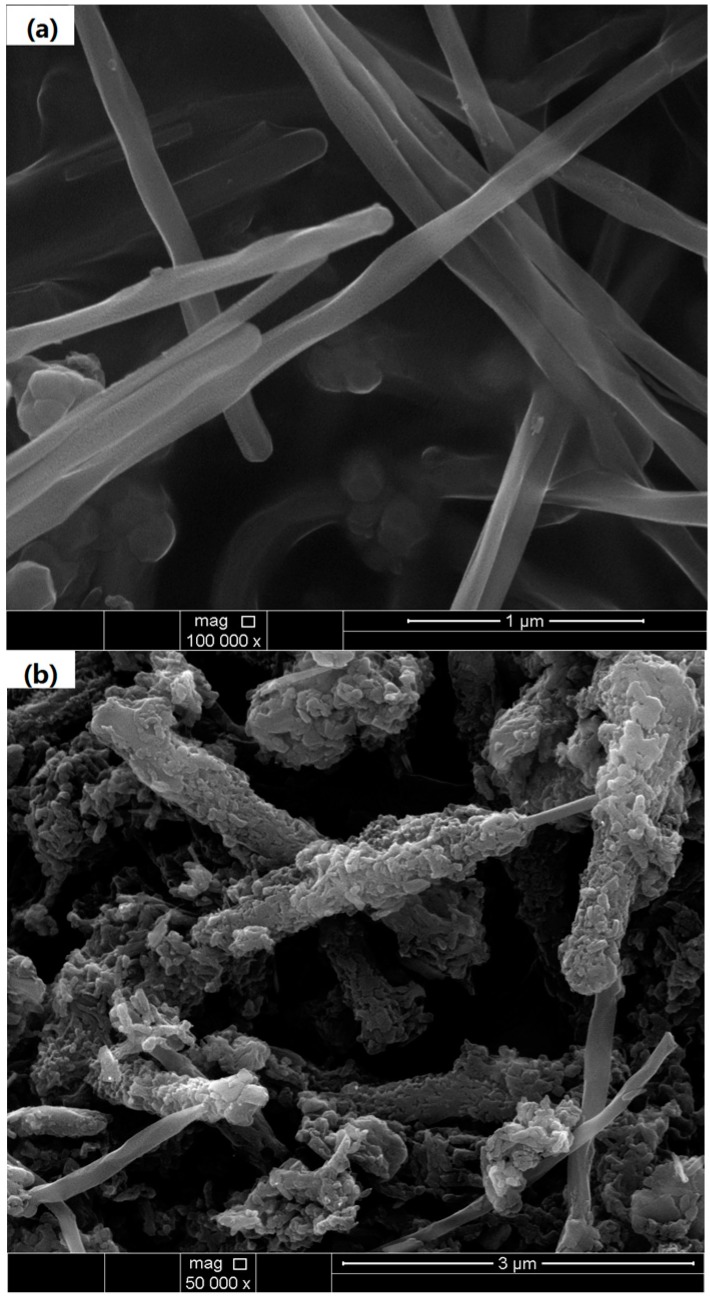
SEM of MWCNTs (**a**) and PANI/F-MWCNTs (**b**).

Figure 1 shows the morphology of the MWCNTs and PANI/F-MWCNTs. The MWCNTs is of cylindrical morphology and it has a smooth surface (Figure 1a). However, the PANI/F-MWCNTs were very rough on the surface, and this apparent difference was ascribed to the F-MWCNTs was covered by PANI. Furthermore, some part of the F-MWCNTs without cladding by the PANI was observed, and the PANI/F-MWCNTs had formed a 3D conductive network by overlapping each other (Figure 1b).

**Figure 2:**
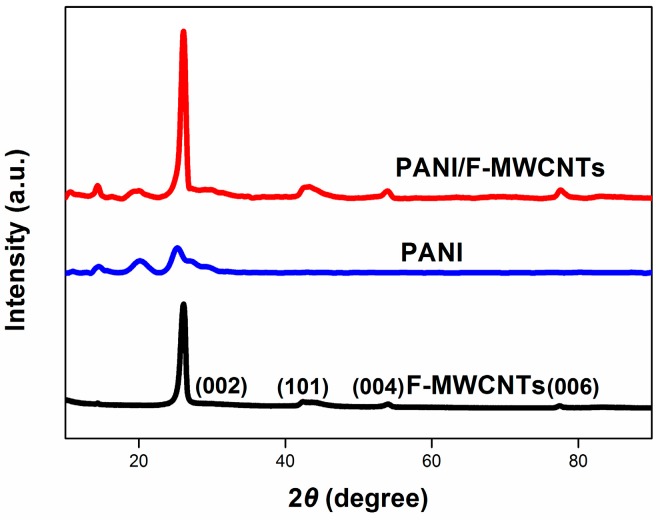
XRD patterns of PANI/F-MWCNTs, PANI, and F-MWCNTs.

The structure of PANI/F-MWCNTs was investigated by XRD analysis. As can be seen in Figure 2, the four diffraction peaks of MWCNTs at 25.6°, 43.2°, 52.8°, and 78.5° could be indexed as (002), (101), (004), and (006) reflection, respectively [24,25]. The same diffraction peaks also emerged in the PANI/F-MWCNTs, suggesting that the crystalline structure of MWCNTs was not destroyed after the compositing process. Furthermore, the reappearance of all the diffraction peaks of PANI in the pattern of PANI/F-MWCNTs confirmed the existence of PANI in the composite electrode.

**Figure 3:**
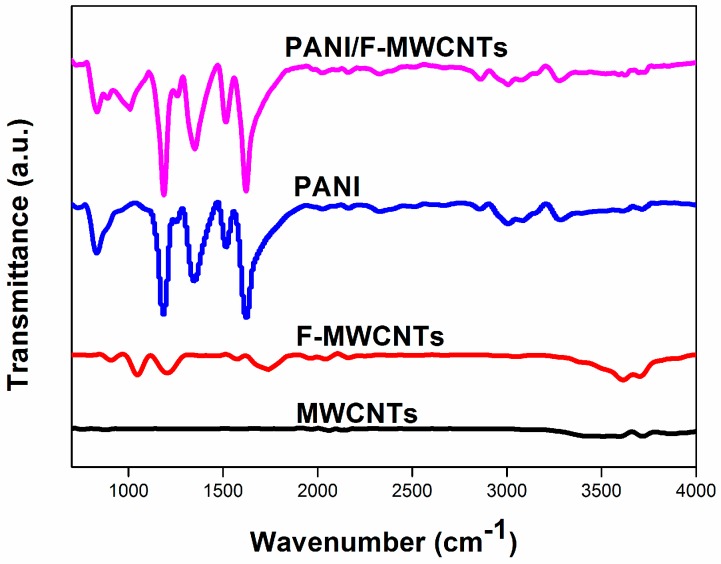
FTIR spectra of PANI/F-MWCNTs, PANI, F-MWCNTs, and MWCNTs.

The chemical composition of PANI/F-MWCNTs was confirmed by FTIR measurements. As shown in Figure 3, compared to the spectrogram of the untreated MWCNTs with 3600 cm^−1^ peak to –OH groups, the adsorption peaks at 1043 cm^−1^ correspond to the C–O bond, as well as 1208 cm^−1^, and 1723 cm^−1^ peak corresponds to the C=O bond of F-MWCNTs was observed, suggesting that the F-MWCNTs had been modified by the –COOH group effectively [5]. The peak at 1340 cm^−1^ could be assigned to the C–N stretching vibration of the benzenoid ring, and the peak at 1180 cm^−1^ is attributed to the aromatic C–H in-plane bending. The peaks centered at 1520 cm^−1^ and 1620 cm^−1^ are due to the C=C and C=N stretching of the benzenoid and quinoid rings, respectively [26,27]. All of the mentioned typical absorption peaks appear in the spectrogram of the PANI/F-MWCNTs confirm the existence of the F-MWCNTs and the PANI, manifesting that the F-MWCNTs had been composited with PANI effectively.

**Figure 4:**
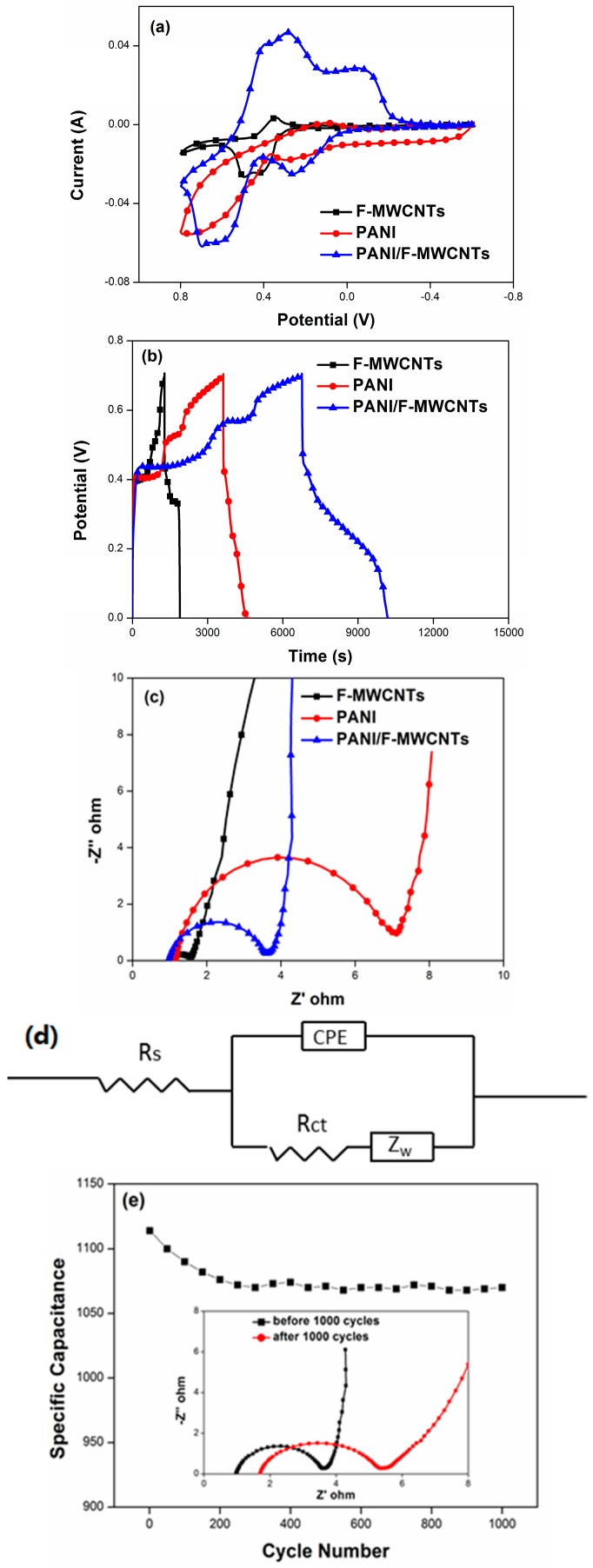
Electrochemical test results of PANI/A-MWCNTs, PANI, and A-MWCNTs in 2 M HQ-1 M H_2_SO_4_ electrolytes. (**a**) CV results at a scan rate of 5 mV/s; (**b**) GCD results at a current density of 0.5 A/g; (**c**) EIS results; (**d**) equivalent circuit; and (**e**) cycling stability of PANI/A-MWCNTs at a current density of 10 A/g; Inset: Nyquist plot of PANI/A-MWCNTs before and after cycling stability test.

Figure 4a shows the CV curves of PANI/F-MWCNTs, PANI, and F-MWCNTs at a scan rate of 5 mV/s in the mixture of 1 mol/L H_2_SO_4_ and 2 mol/L HQ. The potential range was from −0.6 V to 0.8 V. In terms of the electrochemical performance, among three electrode materials, the F-MWCNTs present the lower end of the scale because the performance of F-MWCNTs is attributed to the electronic double-layer capacitance and the redox of HQ, while the PANI’s electrochemical performance is better than that of the F-MWCNTs, which mainly came from the redox of HQ and pseudocapacitance of bulk polyanilline. Distinctly, pseudocapacitance of bulk polyanilline is larger than that of the electronic double-layer capacitance of the F-MWCNTs, but is still limited by shortages in the specific surface area and active situation of the bulk polyanilline. Unlike CV curves of the F-MWCNTs and bulk polyaniline, the PANI/F-MWCNTs show two pair of redox peaks, the peaks at −0.08 V and 0.26 V are the redox pair of PANI and the peaks cantered at 0.28 V and 0.7 V are due to the redox transformation of the HQ. This is why the electrochemical performance of the PANI/F-MWCNTs is situated on the upper of the scale. Furthermore, predominance of the PANI/F-MWCNTs on structure promotes the enhancement of performance of SC. The PANI/F-MWCNTs connected together randomly to build a consecutive 3D conductivity network. The transfer of electric charge and the remove of solvated ions through the 3D conductivity network become more efficiently, consequently, redox in both PANI/F-MWCNTs electrode and HQ acid aqueous electrolyte are developed fully.

Figure 4b shows the galvanostatic discharge curves of PANI/F-MWCNTs, PANI, and F-MWCNTs in the mixture of 1 mol/L H_2_SO_4_ and 2 mol/L HQ at a current density of 1 A/g. the PANI/F-MWCNTs exhibit the best discharge property among these three materials, similar to the result from the CV curves. Owing to nonlinear discharge curves, the galvanostatic capacitances are calculated with Equation (1) using the integral current area of the discharge curve. The specific capacity of PANI/F-MWCNTs is 4002 F/g, which is almost three times larger than that of the pure PANI (1419 F/g) and five times larger than that of the F-MWCNTs (774 F/g). As a comparison, the specific capacitances of the PANI/F-MWCNTs in the HQ-H_2_SO_4_ electrolyte system in this paper and that of the other electrochemical systems reported in the past are listed in Table 1.

Figure 4c,d are respectively the Nyquist plots of the electrochemical impedance spectroscopy (EIS) analysis and equivalent circuit. The semicircle in the high frequency shows the difficulty of the ionic’s exchange during the Faradic process which is generated at the interface of the electrode and electrolyte. The solution resistances of the three systems could be read from the first point of intersection between the semicircle and the *x*-axis. The values of the PANI/F-MWCNT, PANI, and F-MWCNT are 1.04, 1.15, and 1.08 Ω·cm^2^ respectively. They are almost the same, as they were tested in the same electrolyte solution environment. The charge-transfer resistance of PANI/F-MWCNTs, PANI and F-MWCNT from the diameter of the semicircle is 2.66, 6.04, and 0.474 Ω·cm^2^, respectively. The lower charge-transfer resistance means the higher efficiency of the electrode. The introduction of the F-MWCNT effectively reduces the charge-transfer resistance of the PANI-based electrode. Furthermore, the potential drop (iR_drop_) at the beginning of the discharge process (Figure 4b) is the total resistance of the electrochemical system, including the internal resistance of the electrode, the electrical connection resistance, the bulk solution resistance, and the resistance of ion migration in the electrode material. According to the potential drop, the equivalent series resistance (ESR) of the system could be calculated by Equation (2). The ESR of PANI/F-MWCNTs, PANI, and F-MWCNTs are 26, 33, and 29 Ω·cm^2^, respectively. The ESR of PANI/F-MWCNTs is the lowest among three electrodes in this present study, suggesting an excellent electrochemical performance. Reasonably, the electrochemical performance depends not only on the synergistic effect between the PANI and the HQ solution, but also the 3D conductive network with good electrical conductivity and short route of ionic removal.

The cycling stability of PANI/F-MWCNTs was tested under a current density of 10 A/g in a HQ (2 mol/L)-H_2_SO_4_ (1 mol/L) electrolyte, as shown in Figure 4e. The discharge specific capacitance of the PANI/F-MWCNTs still keeps 948 F/g after charge-discharge 1000 cycles, and the retention of capacitance is 96%. Furthermore, the charge-transfer resistance of PANI/F-MWCNTs had almost not changed during the charge-discharge process. After the charge-discharge of 1000 cycles, the charge-transfer resistance was only 1.005 Ω·cm^2^ larger than pristine.

**Figure 5:**
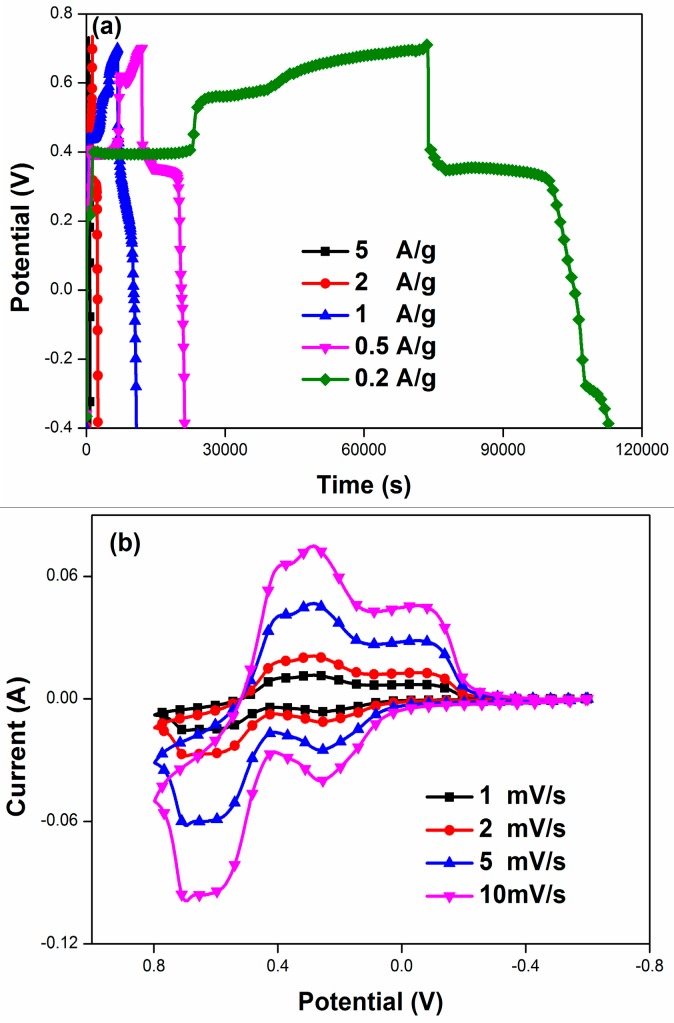
Electrochemical test results of PANI/A-MWCNTs in 2 M HQ-1 M H_2_SO_4_ electrolytes. (**a**) Specific capacitance at different current density; and (**b**) CV results at different scan rates.

The rate capacity of PANI/F-MWCNTs was tested with different current densities (0.2, 0.5, 1, 2, and 5 A/g) in 2 M HQ-1 M H_2_SO_4_ electrolytes. As can be seen in the Figure 5a, the specific capacitance of PANI/F-MWCNTs increases with the declining of the current density. The maximum specific capacitance could get to 7926 F/g when the current density is 0.2 A/g. At the various current density, the electrochemical system exhibits a serious high specific capacitance: 7926 F/g (0.2 A/g), 5375 F/g (0.5 A/g), 4002 F/g (1.0 A/g), 2596 F/g (2.0 A/g), as well as 1620 F/g (5.0 A/g). The specific capacitance declines about five times when the current density is 25 times larger than before, which suggests an excellent high-rate discharge ability of the PANI/F-MWCNTs. Figure 5b shows the electrochemical process between PANI/F-MWCNTs and HQ (2 mol/L) in 1 mol/L H_2_SO_4_ by a CV curves with various scan rates (1, 2, 5, and 10 mV/s). In detail, with the increase of the scan rate, the peak current is enhanced and the separation of the peak potentials is enlarged slightly. Significantly, in this CV curve at a high scan rate, the phenomenon of two-pair redox peaks concomitance with the largest CV area indicate that the coexistence of the redox of both of HQ and polyaniline in an electrochemical process can greatly promote the development of the synergistic effect.

**Figure 6:**
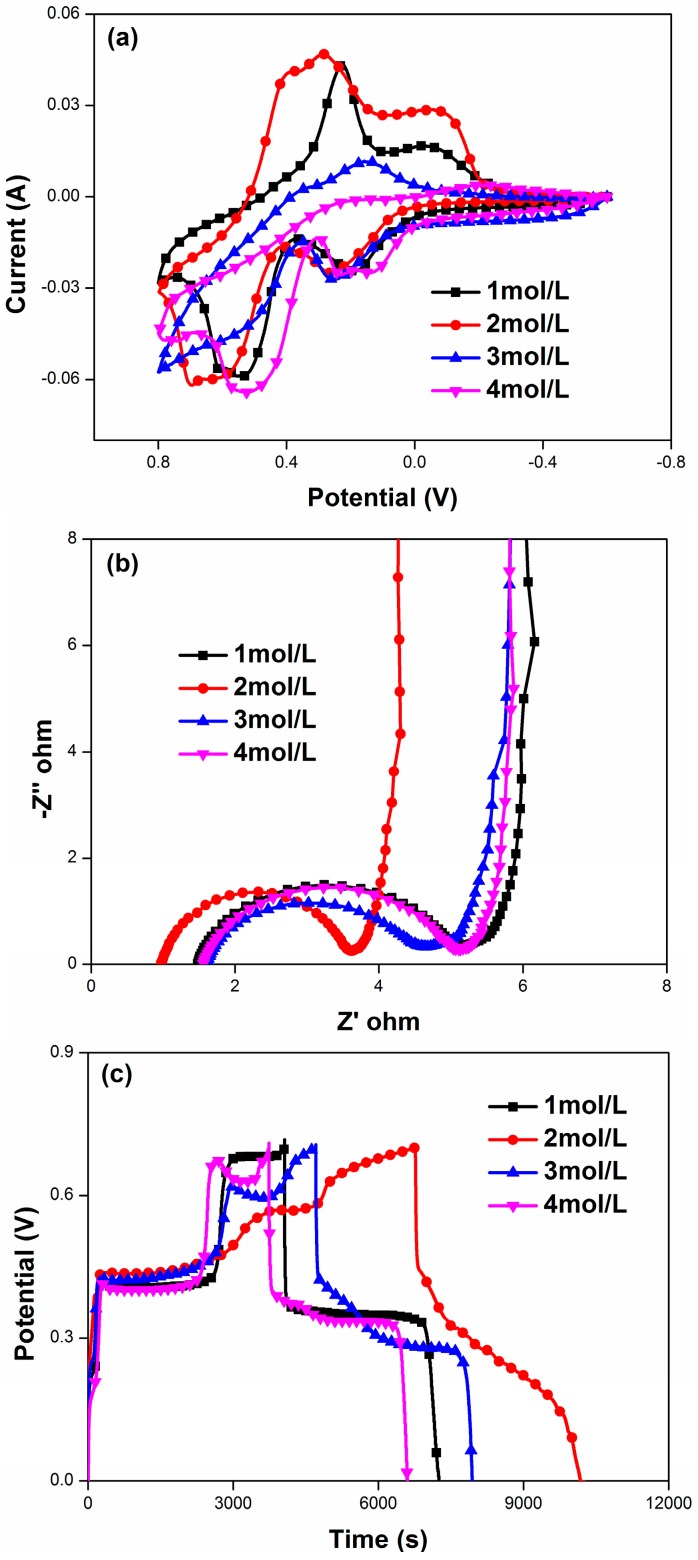
The CV (**a**); EIS (**b**); and GCD (**c**) results of PANI/A-MWCNTs in 1 M H_2_SO_4_ with different concentrations of HQ.

The effect of concentration of HQ on the specific capacitance is presented by CV curves (5 mV/s) in the Figure 6a. The optimal concentration of HQ in which the largest peak current and CV area visualize is 2 mol/L HQ, larger or smaller than this concentration, the area of CV curves and current peak value will decrease. Obviously, low concentration of the HQ lead to the decline of the ionic conductivity, and thus the deficient number of ion to meet the number of redox sites of the PANI/F-MWCNTs, as a result, the oxidation-reduction reaction both in electrolyte and electrode could not fully accomplish. Otherwise, a high concentration of HQ is not conducive to the enhancement of specific capacitance due to the separating out of HQ crystals from the solution and their impeditive effect on ionic or electric transfer during the electrochemical process. As shown in Figure 6b, the above-mentioned results of CV measurement agree well with the EIS analysis of the PANI/F-MWCNTs in various concentrations of HQ. Typically, when the concentration of HQ is 2 mol/L, the PANI/F-MWCNTs possess the smallest solution resistances and the lowest charge-transfer resistance. Similarly, in the Figure 6c, the highest capacitance from the discharge process also is achieved at this optimal HQ concentration. The effect of concentration of HQ on performances, such as specific capacitances, and values of Rs and Rct [46], have been listed in Table 2.

**Figure 7:**
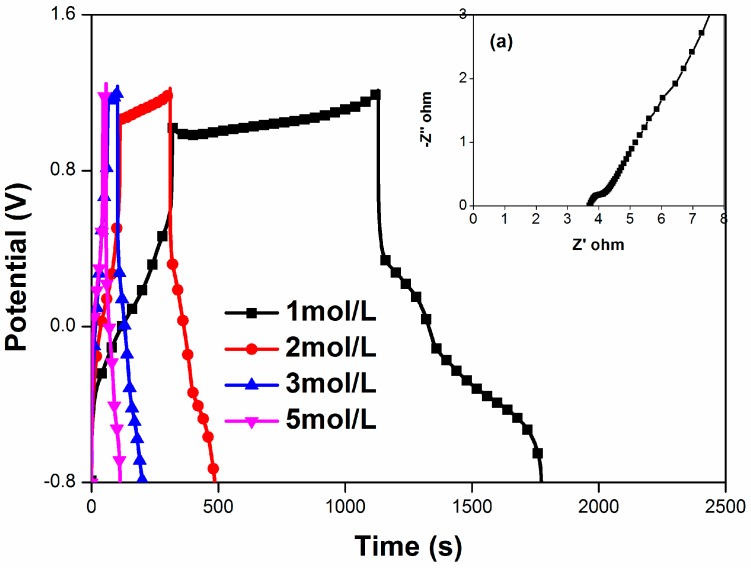
GCD and EIS (inset Figure 7a) curves of PANI/A-MWCNTs in a two-electrode system.

In order to fully explore the performance of our supercapacitor, the present work investigated the electrochemical performance with a two-electrode symmetric electrochemical system. Figure 7 shows the charging–discharging curves of PANI/F-MWCNTs at different current density (1, 2, 3, and 5 A/g), and the EIS analysis (Figure 7a, inset) in 1 mol/L H_2_SO_4_–2 mol/L HQ. The charge-transfer resistance of 0.54 Ω·cm^2^ suggests good electric conductivity and efficient charge transfer of this system. The discharge specific capacitances at various current densities are 205 F/g (1 A/g) and 119 F/g (5 A/g), respectively. The energy density and the power density are 114 Wh/kg and 805 W/kg, respectively, in this symmetrical electrode system at 1 A/g. It is important to note that the mass which is used to calculate the energy density and the power density is the total mass of electrode, including PANI/F-MWCNTs composites (80 wt. %), PVDF (10 wt. %), and acetylene black (10 wt. %).

## 5. Conclusions

In a summary, the structure of PANI/F-MWCNTs composites is characterized by SEM, XRD, and FTIR, The F-MWCNTs covered by the PANI build 3D conductive networks to increase the synergetic redox effect in both the composite electrode and the HQ electrolyte of the SC. The concentration of the HQ effect on performance of the SC is investigated, and the optimal concentration of the HQ for the enhancement of performance is determined by a serious electrochemical measurement. The highest specific capacitance from the GCD measurement is 7926 F/g at 0.2 A/g, a new record in the performance of SC. The maximum energy density reaches 114 Wh/kg in a two-electrode symmetric electrochemical system at 1 A/g in 2 M HQ-1 M H_2_SO_4_ solution. In addition, the specific capacitance retention is 96% after 1000 galvanostatic charge/discharge cycles, indicating an excellent cyclic stability.

## Figures and Tables

**Table 1:** Comparison of specific capacitance between the system of our present works and the reported values.

Materials	Electrolytes	Test Condition	Specific Capacitance （F/g）	References
KSC/NCNTs/PANI	2 M H_2_SO_4_	0.1 A/g	1090	[28]
Px-MWCNT	1 M H_2_SO_4_	20 mA/cm^2^	118	[29]
PANI/MWCNT	1 M NaNO_3_	0.5 A/g	176.5	[30]
PANI/CNTf	1 M H_2_SO_4_	2 A/g	1744	[31]
PANI/SWCNT	1 M H_2_SO_4_	5 mA/cm^2^	485	[32]
PANI/MWCNTs/rGO	1 M H_2_SO_4_	0.5 A/g	987	[33]
PANI/MWNT	1 M H_2_SO_4_	5 mV/s	139	[34]
PANI/MWNTs	1 M NaNO_3_	5 mA/cm^2^	328	[35]
MWCNT/PANI	0.5 M H_2_SO_4_	5 mA/cm^2^	500	[36]
PANI/MWCNT	0.1 M H_2_SO_4_	5 mV/s	560	[37]
Ag-PANI/MWCNTs	1 M KCl	1 A/g	528	[38]
PANI-g-TCNF	1 M H_2_SO_4_	0.3 A/g	550	[39]
PANI/sMWCNT	1 M H_2_SO_4_	1 A/g	431.3	[40]
Activated Charcoal/HQ	1 M H_2_SO_4_	5 mV/s	207	[41]
Activated carbon	C_6_H_4_Br_2_O_2_-2 M KOH	5 mV/s	314	[42]
Activated carbon	PVA-H_2_SO_4_-HQ/ PVA-H_2_SO_4_-MB	0.5 A/g	563.7	[43]
PANI/SnO2	0.4 M HQ-1 M H_2_SO_4_	0.5 A/g	857	[44]
PANI	0.4 M HQ-1 M H_2_SO_4_	0.5 A/g	584	[45]
PANI/F-MWCNTs	1 M H_2_SO_4_-2 M HQ	5 A/g	1620	Present work
		2 A/g	2596	
		1 A/g	4002	
		0.5 A/g	5375	
		0.2 A/g	7926	

**Table 2:** The specific capacitance, electrolyte resistance, and charge-transfer resistance at different HQ concentration.

HQ Concentration	C (F/g)	Rs (Ω·cm^2^)	Rct (Ω·cm^2^)
1	3927	1.48	3.76
2	4002	0.975	2.642
3	3846	1.61	3.04
4	3559	1.56	3.61

## References

[1] Yang Z., Ren J., Zhang Z., Chen X., Guan G., Qiu L., Zhang Y., Peng H. (2015). Recent Advancement of Nanostructured Carbon for Energy Applications. Chem. Rev..

[2] Yun Y.S., Park H.H., Jin H.-J. (2012). Pseudocapacitive Effects of N-Doped Carbon Nanotube Electrodes in Supercapacitors. Materials.

[3] Lee H.-M., Lee K., Kim C.-K. (2014). Electrodeposition of Manganese-Nickel Oxide Films on a Graphite Sheet for Electrochemical Capacitor Applications. Materials.

[4] Zu L., Cui X., Jiang Y., Hu Z., Lian H., Liu Y., Jin Y., Li Y., Wang X. (2015). Preparation and Electrochemical Characterization of Mesoporous Polyaniline-Silica Nanocomposites as an Electrode Material for Pseudocapacitors. Materials.

[5] Wang J.-G., Kang F., Wei B. (2015). Engineering of MnO_2_-based nanocomposites for high-performance supercapacitors. Prog. Mater. Sci..

[6] Cao Z., Wei B. (2013). A perspective: Carbon nanotube macro-films for energy storage. Energy Environ. Sci..

[7] Zhong C., Deng Y., Hu W., Qiao J., Zhang L., Zhang J. (2015). A review of electrolyte materials and compositions for electrochemical supercapacitors. Chem. Soc. Rev..

[8] Aravindan V., Gnanaraj J., Lee Y.-S., Madhavi S. (2014). Insertion-Type Electrodes for Nonaqueous Li-Ion Capacitors. Chem. Rev..

[9] Tsai Y.-C., Yang W.-D., Lee K.-C., Huang C.-M. (2016). An Effective Electrodeposition Mode for Porous MnO_2_/Ni Foam Composite for Asymmetric Supercapacitors. Materials.

[10] Salunkhe R.R., Tang J., Kamachi Y., Nakato T., Kim J.H., Yamauchi Y. (2015). Asymmetric Supercapacitors Using 3D Nanoporous Carbon and Cobalt Oxide Electrodes Synthesized from a Single Metal-Organic Framework. ACS Nano.

[11] Fic K., Lota G., Meller M., Frackowiak E. (2012). Novel insight into neutral medium as electrolyte for high-voltage supercapacitors. Energy Environ. Sci..

[12] Béguin F., Presser V., Balducci A., Frackowiak E. (2014). Carbons and Electrolytes for Advanced Supercapacitors. Adv. Mater..

[13] Senthilkumar S.T., Selvan R.K., Melo J.S. (2013). Redox additive/active electrolytes: A novel approach to enhance the performance of supercapacitors. J. Mater. Chem. A.

[14] Lota G., Frackowiak E. (2009). Striking capacitance of carbon/iodide interface. Electrochem. Commun..

[15] Li Q., Li K., Sun C., Li Y. (2007). An investigation of Cu^2+^ and Fe^2+^ ions as active materials for electrochemical redox supercapacitors. J. Electroanal. Chem..

[16] Wang X., Chandrabose R.S., Chun S.-E., Zhang T., Evanko B., Jian Z., Boettcher S.W., Stucky G.D., Ji X. (2015). High Energy Density Aqueous Electrochemical Capacitors with a KI-KOH Electrolyte. ACS Appl. Mater. Interfaces.

[17] Mai L.Q., Minhas-Khan A., Tian X., Hercule K.M., Zhao Y.L., Lin X., Xu X. (2013). Synergistic interaction between redox-active electrolyte and binder-free functionalized carbon for ultrahigh supercapacitor performance. Nat. Commun..

[18] Roldán S., Blanco C., Granda M., Menéndez R., Santamaría R. (2011). Towards a Further Generation of High-Energy Carbon-Based Capacitors by Using Redox-Active Electrolytes. Angew. Chem. Int. Ed..

[19] Roldán S., Granda M., Menéndez R., Santamaría R., Blanco C. (2011). Mechanisms of Energy Storage in Carbon-Based Supercapacitors Modified with a Quinoid Redox-Active Electrolyte. J. Phys. Chem. C.

[20] Jiang Y., Cui X., Zu L., Hu Z., Gan J., Lian H., Liu Y., Xing G. (2015). High Rate Performance Nanocomposite Electrode of Mesoporous Manganese Dioxide/Silver Nanowires in KI Electrolytes. Nanomaterials.

[21] Hu Z., Zu L., Jiang Y., Lian H., Liu Y., Li Z., Chen F., Wang X., Cui X. (2015). High Specific Capacitance of Polyaniline/Mesoporous Manganese Dioxide Composite Using KI-H_2_SO_4_ Electrolyte. Polymers.

[22] Hu Z., Zu L., Jiang Y., Lian H., Liu Y., Wang X., Cui X. (2015). High performance nanocomposite electrodes of mesoporous silica platelet-polyaniline synthesized via impregnation polymerization. Polym. Compos..

[23] Abdulla S., Mathew T.L., Pullithadathil B. (2015). Highly sensitive, room temperature gas sensor based on polyaniline-multiwalled carbon nanotubes (PANI/MWCNTs) nanocomposite for trace-level ammonia detection. Sens. Actuators B Chem..

[24] Chen X., Li H., Wu H., Wu Y., Shang Y., Pan J., Xiong X. (2016). Fabrication of TiO_2_@PANI nanobelts with the enhanced absorption and photocatalytic performance under visible light. Mater. Lett..

[25] Sk M.M., Yue C.Y., Jena R.K. (2015). Non-covalent interactions and supercapacitance of pseudo-capacitive composite electrode materials (MWCNTCOOH/MnO_2_/PANI). Synth. Met..

[26] Belin T., Epron F. (2005). Characterization methods of carbon nanotubes: A review. Mater. Sci. Eng. B.

[27] Youssef A.M., Mohamed S.A., Abdel-Aziz M.S., Abdel-Aziz M.E., Turky G., Kamel S. (2016). Biological studies and electrical conductivity of paper sheet based on PANI/PS/Ag-NPs nanocomposite. Carbohydr. Polym..

[28] Lu X., Hu Y., Wang L., Guo Q., Chen S., Chen S., Hou H., Song Y. (2016). Macroporous Carbon/Nitrogen-doped Carbon Nanotubes/Polyaniline Nanocomposites and Their Application in Supercapacitors. Electrochim. Acta.

[29] Potphode D.D., Sivaraman P., Mishra S.P., Patri M. (2015). Polyaniline/partially exfoliated multi-walled carbon nanotubes based nanocomposites for supercapacitors. Electrochim. Acta.

[30] Lee S.-Y., Kim J.-I., Park S.-J. (2014). Activated carbon nanotubes/polyaniline composites as supercapacitor electrodes. Energy.

[31] Bavio M.A., Acosta G.G., Kessler T. (2014). Synthesis and characterization of polyaniline and polyaniline—Carbon nanotubes nanostructures for electrochemical supercapacitors. J. Power Sources.

[32] Gupta V., Miura N. (2006). Polyaniline/single-wall carbon nanotube (PANI/SWCNT) composites for high performance supercapacitors. Electrochim. Acta.

[33] Tran V.C., Nguyen V.H., Nguyen T.T., Lee J.H., Huynh D.C., Shim J.-J. (2016). Polyaniline and multi-walled carbon nanotube-intercalated graphene aerogel and its electrochemical properties. Synth. Met..

[34] Yoon S.-B., Yoon E.-H., Kim K.-B. (2011). Electrochemical properties of leucoemeraldine, emeraldine, and pernigraniline forms of polyaniline/multi-wall carbon nanotube nanocomposites for supercapacitor applications. J. Power Sources.

[35] Dong B., He B.-L., Xu C.-L., Li H.-L. (2007). Preparation and electrochemical characterization of polyaniline/multi-walled carbon nanotubes composites for supercapacitor. Mater. Sci. Eng. B.

[36] Zhang J., Kong L.-B., Wang B., Luo Y.-C., Kang L. (2009). In-situ electrochemical polymerization of multi-walled carbon nanotube/polyaniline composite films for electrochemical supercapacitors. Synth. Met..

[37] Zhou Y., Qin Z.-Y., Li L., Zhang Y., Wei Y.-L., Wang L.-F., Zhu M.-F. (2010). Polyaniline/multi-walled carbon nanotube composites with core–shell structures as supercapacitor electrode materials. Electrochim. Acta.

[38] Dhibar S., Das C.K. (2014). Silver Nanoparticles Decorated Polyaniline/Multiwalled Carbon Nanotubes Nanocomposite for High-Performance Supercapacitor Electrode. Ind. Eng. Chem. Res..

[39] Kotal M., Thakur A.K., Bhowmick A.K. (2013). Polyaniline-carbon nanofiber composite by a chemical grafting approach and its supercapacitor application. ACS Appl. Mater. Interfaces.

[40] Sun M., Wang G., Li X., Cheng Q., Li C. (2012). Interfacial Synthesis and Supercapacitive Performance of Hierarchical Sulfonated Carbon Nanotubes/Polyaniline Nanocomposites. Ind. Eng. Chem. Res..

[41] Singh C., Paul A. (2015). Physisorbed Hydroquinone on Activated Charcoal as a Supercapacitor: An Application of Proton-Coupled Electron Transfer. J. Phys. Chem. C.

[42] Gastol D., Walkowiak J., Fic K., Frackowiak E. (2016). Enhancement of the carbon electrode capacitance by brominated hydroquinones. J. Power Sources.

[43] Zhong J., Fan L.-Q., Wu X., Wu J.-H., Liu G.-J., Lin J.-M., Huang M.-L., Wei Y.-L. (2015). Improved energy density of quasi-solid-state supercapacitors using sandwich-type redox-active gel polymer electrolytes. Electrochim. Acta.

[44] Zhu Y., Liu E., Luo Z., Hu T., Liu T., Li Z., Zhao Q. (2014). A hydroquinone redox electrolyte for polyaniline/SnO_2_ supercapacitors. Electrochim. Acta.

[45] Xie H., Zhu Y., Wu Y., Wu Z., Liu E. (2014). The effect of hydroquinone as an electrolyte additive on electrochemical performance of the polyaniline supercapacitor. Mater. Res. Bull..

[46] Yu H., Wu J., Fan L., Xu K., Zhong X., Lin Y., Lin J. (2011). Improvement of the performance for quasi-solid-state supercapacitor by using PVA–KOH–KI polymer gel electrolyte. Electrochim. Acta.

